# Mutation of Phenylalanine 23 of Newcastle Disease Virus Matrix Protein Inhibits Virus Release by Disrupting the Interaction between the FPIV L-Domain and Charged Multivesicular Body Protein 4B

**DOI:** 10.1128/spectrum.04116-22

**Published:** 2023-01-25

**Authors:** Yu Pei, Jia Xue, Qingyuan Teng, Delan Feng, Min Huang, Rong Liang, Xiao Li, Ye Zhao, Jing Zhao, Guozhong Zhang

**Affiliations:** a Key Laboratory of Animal Epidemiology of the Ministry of Agriculture, College of Veterinary Medicine, China Agricultural University, Beijing, China; Barnard College, Columbia University

**Keywords:** Newcastle disease virus, M protein, FPIV L-domain, virus release, ESCRT machinery, CHMP4B

## Abstract

The matrix (M) protein FPIV L-domain is conserved among multiple paramyxoviruses; however, its function and the associated mechanism remain unclear. In this study, the paramyxovirus Newcastle disease virus (NDV) was employed to study the FPIV L-domain. Two recombinant NDV strains, each carrying a single amino acid mutation at the Phe (F23) or Pro (P24) site of ^23^FPIV/I^26^ L-domain, were rescued. Growth defects were observed in only the recombinant SG10-F23A (rSG10-F23A) strain. Subsequent studies focused on rSG10-F23A revealed that the virulence, pathogenicity, and replication ability of this strain were all weaker than those of wild-type strain rSG10 and that a budding deficiency contributed to those weaknesses. To uncover the molecular mechanism underlying the rSG10-F23A budding deficiency, the bridging proteins between the FPIV L-domain and endosomal sorting complex required for transported (ESCRT) machinery were explored. Among 17 candidate proteins, only the charged multivesicular body protein 4 (CHMP4) paralogues were found to interact more strongly with the NDV wild-type M protein (M-WT) than with the mutated M protein (M-F23A). Overexpression of M-WT, but not of M-F23A, changed the CHMP4 subcellular location to the NDV budding site. Furthermore, a knockdown of CHMP4B, the most abundant CHMP4 protein, inhibited the release of rSG10 but not that of rSG10-F23A. From these findings, we can reasonably infer that the F23A mutation of the FPIV L-domain blocks the interaction between the NDV M protein and CHMP4B and that this contributes to the budding deficiency and consequent growth defects of rSG10-F23A. This work lays the foundation for further study of the FPIV L-domain in NDV and other paramyxoviruses.

**IMPORTANCE** Multiple viruses utilize a conserved motif, termed the L-domain, to act as a cellular adaptor for recruiting host ESCRT machinery to their budding site. Despite the FPIV type L-domain having been identified in some paramyxoviruses 2 decades ago, its function in virus life cycles and its method of recruiting the ESCRT machinery are poorly understood. In this study, a single amino acid mutation at the F23 site of the ^23^FPIV^26^ L-domain was found to block NDV budding at the late stage. Furthermore, CHMP4B, a core component of the ESCRT-III complex, was identified as a main factor that links the FPIV L-domain and ESCRT machinery together. These results extend previous understanding of the FPIV L-domain and, therefore, not only provide a new approach for attenuating NDV and other paramyxoviruses but also lay the foundation for further study of the FPIV L-domain.

## INTRODUCTION

Paramyxoviruses such as Nipah virus (NiV), Hendra virus (HeV), mumps virus (MuV), measles virus (MeV), canine distemper virus (CDV), feline morbillivirus (FeMV), and Newcastle disease virus (NDV), which are single-stranded negative-sense RNA viruses, can affect both humans and animals and always cause severe respiratory and gastrointestinal diseases. A paramyxovirus epidemic would impose severe burdens on human health and livestock industries (1–4).

Paramyxovirus M protein, which has been found to be a nucleocytoplasmic trafficking protein (5), plays multiple roles in the viral life cycle. Generally, the functions of M protein can be summarized into three roles. First, in the early stage of paramyxovirus infection, M protein usually traffics into the nucleus via its nuclear location signal and transiently aggregates in the nucleolus, and this aggregation of M protein of respiratory syncytial virus (RSV), NDV, and MeV was reported to affect the transcription, synthesis, and posttranscriptional modification of host proteins (6–8). Second, M proteins of some paramyxoviruses can participate in the innate immune evasion process. For example, the NiV M protein participates in suppressing the host antiviral type I interferon (IFN-I) response by targeting TRIM6 (9). And the human parainfluenza virus type 3 (HPIV3) M protein interacts with both the TUFM and LC3 proteins, which links autophagosomes and mitochondria and leads to inhibition of IFN-I response (10). Third, M protein plays a central role in the assembly and budding process of progeny virions and virus-like particles (VLPs). Paramyxovirus M proteins rely mainly on two kinds of driving force to manipulate viral release. Dimers of M protein can form arrays on the inner surface of the plasma membrane and promote the curvature of it (11, 12). Alternatively, some conserved peptides on M protein, called the L-domain, can utilize the host endosomal sorting complex required for transported (ESCRT) machinery to facilitate paramyxovirus release; however, even a single amino acid mutation in this domain causes a severe budding deficiency in paramyxoviruses (13–15).

ESCRT machinery, first discovered in yeast (16), is responsible for the processes of cellular membrane abscission and remodeling (17). This machinery contains three functional subcomplexes named ESCRT-I, ESCRT-II, and ESCRT-III. ESCRT-I consists of TSG101, VPS37, VPS28, and UBAP1. In the biogenesis of host intraluminal vesicles (ILVs), the ESCRT-II complex is recruited to ESCRT-I via the interaction between VPS28 and the ESCRT-II component EAP45. ESCRT-II is a kind of heterotetramer, formed by one copy of EAP45, one copy of EAP30, and two copies of EAP20. Among these, EAP20 always serves as the bridging factor between ESCRT-II and ESCRT-III in ILV biogenesis by interacting with charged multivesicular body protein 6 (CHMP6). ESCRT-III contains 12 α-helical charged multivesicular body proteins known as CHMP1A, CHMP1B, CHMP2A, CHMP2B, CHMP3, CHMP4A, CHMP4B, CHMP4C, CHMP5, CHMP6, CHMP7, and IST1, and it plays a key role in mediating cellular membrane constriction (17, 18). The ESCRT machinery not only is required in cellular processes like plasma membrane repair but also is hijacked by some viruses in different stages of propagation, especially the budding stage (19–22). In these cases, viruses usually encode some conserved peptides, called the L-domain, as an adaptor to recruit ESCRT-associated proteins and utilize them to catalyze membrane fission events. At present, four kinds of L-domain have been identified: P(T/S)AP (23), YPX_(n)_L (24), PPXY (25), and FPIV (15). Unlike FPIV, the other three L-domains were originally discovered in retroviruses, and their functions have been well expanded. In short, the P(T/S)AP L-domain can recruit TSG101, a component of the ESCRT-I complex, to hijack the ESCRT machinery (26). The YPx_(1, 3)_L L-domain can interact with ALIX, an accessory protein of ESCRT that can recruit CHMP4B directly (27). The PPXY L-domain can bind to the Nedd4 family ubiquitin ligases, a subgroup of HETC-E3 ligases, which can target and initiate ESCRT machinery via their enzymatic activity (28). As for FPIV, which has been found only in paramyxoviruses (15), the identities of any ESCRT-associated proteins it might hijack remain a mystery.

Viruses rely on their host to complete their life cycle. Many studies have demonstrated that the mutation at key amino acid sites on L-domains, which disrupts the interaction between viral proteins and selected ESCRT components, causes severe budding deficiency of the virus (19). Although several paramyxoviruses, including parainfluenza virus 5 (PIV5) (15), MuV (29), and NDV (13), utilize the FPIV L-domain to accomplish their budding process, the main factor of ESCRT machinery that is recruited by the FPIV L-domain is still unknown. In this study, recombinant SG10-F23A (rSG10-F23A) bearing a single amino acid mutation on the F23 site of the ^23^FPIV^26^ L-domain was rescued. Its replication, pathogenicity, and budding efficiency were compared with those of the wild-type (WT) rSG10. Furthermore, CHMP4B, a component of ESCRT-III, was identified as one of the main factors that linked FPIV L-domain and ESCRT machinery. This study provides important insight into the function of the FPIV L-domain in NDV and other paramyxoviruses.

## RESULTS

### Rescue and confirmation of rNDVs.

**FIG 1:**
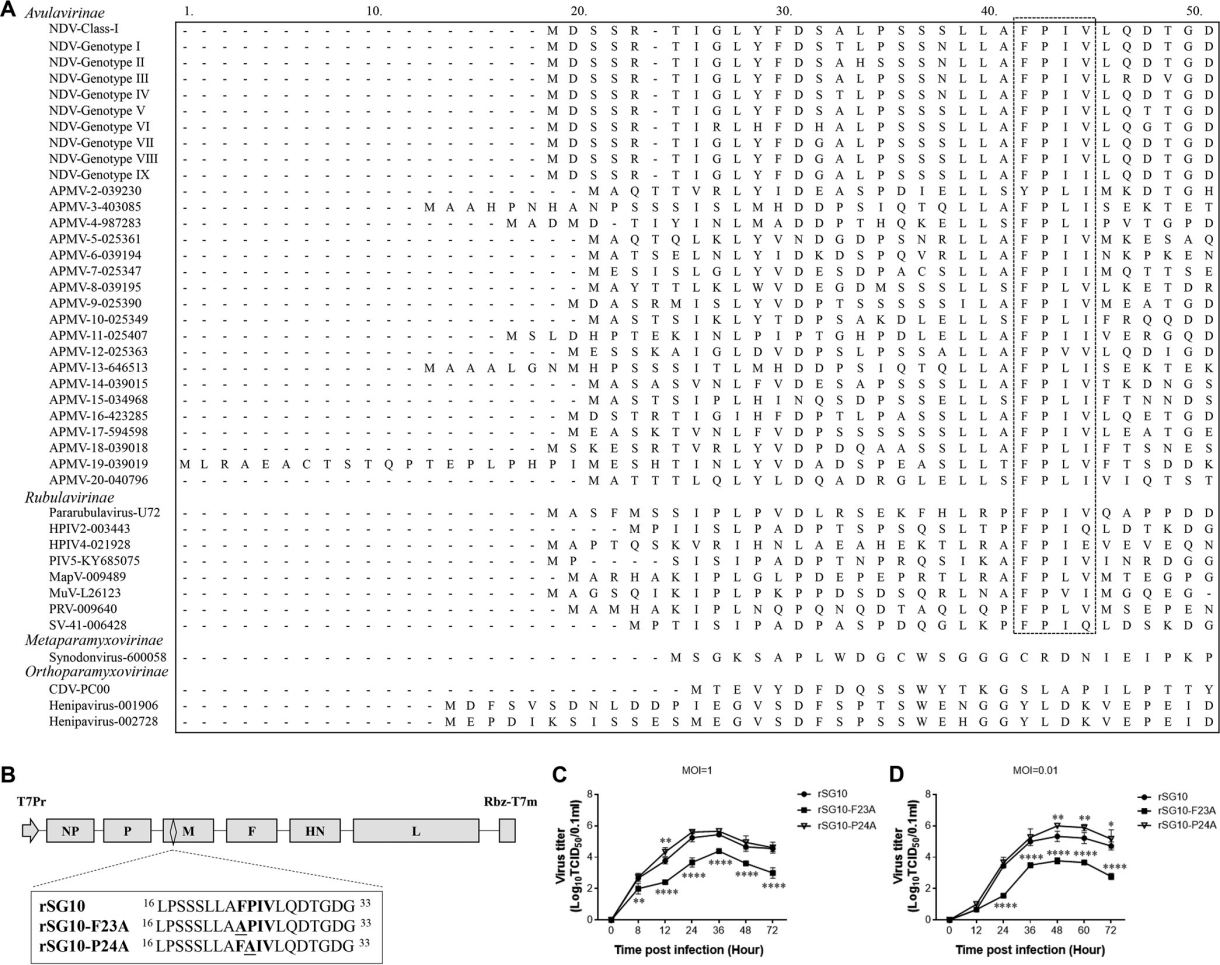
Rescue and confirmation of the rNDVs. (A) Amino acid sequence alignment of the M proteins from the family *Paramyxoviridae*. The conserved FPIV-like sequence is outlined by a black dotted line. (B) Schematic diagram of rSG10. The mutant sites of rSG10-F23A and rSG10-P24A are marked by a black underline. (C and D) One-step (MOI = 1) and multistep (MOI = 0.01) growth kinetics of rSG10, rSG10-F23A, and rSG10-P24A in Vero cells. Cell supernatants from infected cells were collected at the indicated time points, and the viral titers were determined by titration on corresponding cells. *P* values were calculated by two-way ANOVA.

The family *Paramyxoviridae* contains four subfamilies: *Avulavirinae*, *Metaparamyxovirinae*, *Orthoparamyxovirinae*, and *Rubulavirinae*. An amino acid sequence alignment of M proteins from this family revealed that the FPIV-like L-domain is conserved among members of the subfamilies *Avulavirinae* and *Rubulavirinae* (Fig. 1A). In addition, the phenylalanine (F) and proline (P) in this L-domain were more conserved than the other two amino acids. We thus rescued two NDV rSG10 strain-based rNDVs with single amino acid mutations to their M protein, one with the mutation at the F23 site and one with the mutation at the P24 site, and named them rSG10-F23A and rSG10-P24A, respectively (Fig. 1B). The rSG10-P24A strain exhibited growth characteristics similar to those of rSG10, whereas the rSG10-F23A strain exhibited significant growth defects at 24 to 72 h postinfection (hpi) compared with rSG10 according to both one-step and multistep growth curves (Fig. 1C and D). Thus, the rSG10-F23A strain was selected for use in the following virulence and pathogenicity experiments.

### Mutation of the FPIV L-domain affects the virulence and pathogenicity of NDV.

To study the *in vivo* biological characteristics of NDV after FPIV L-domain mutation, we evaluated the virulence of rSG10 and rSG10-F23A with the following two indicators: mean death time (MDT) and intracerebral pathogenicity index (ICPI). The MDT of rSG10-F23A was 96 h, which is longer than the rSG10 MDT of 52.8 h, and the ICPI of rSG10-F23A was 1.58, which is lower than the rSG10 ICPI of 1.74. Collectively, these data demonstrate that the mutation of the FPIV L-domain in rSG10-F23A caused this strain to have reduced virulence compared with that of the wild-type rSG10 strain.

**FIG 2:**
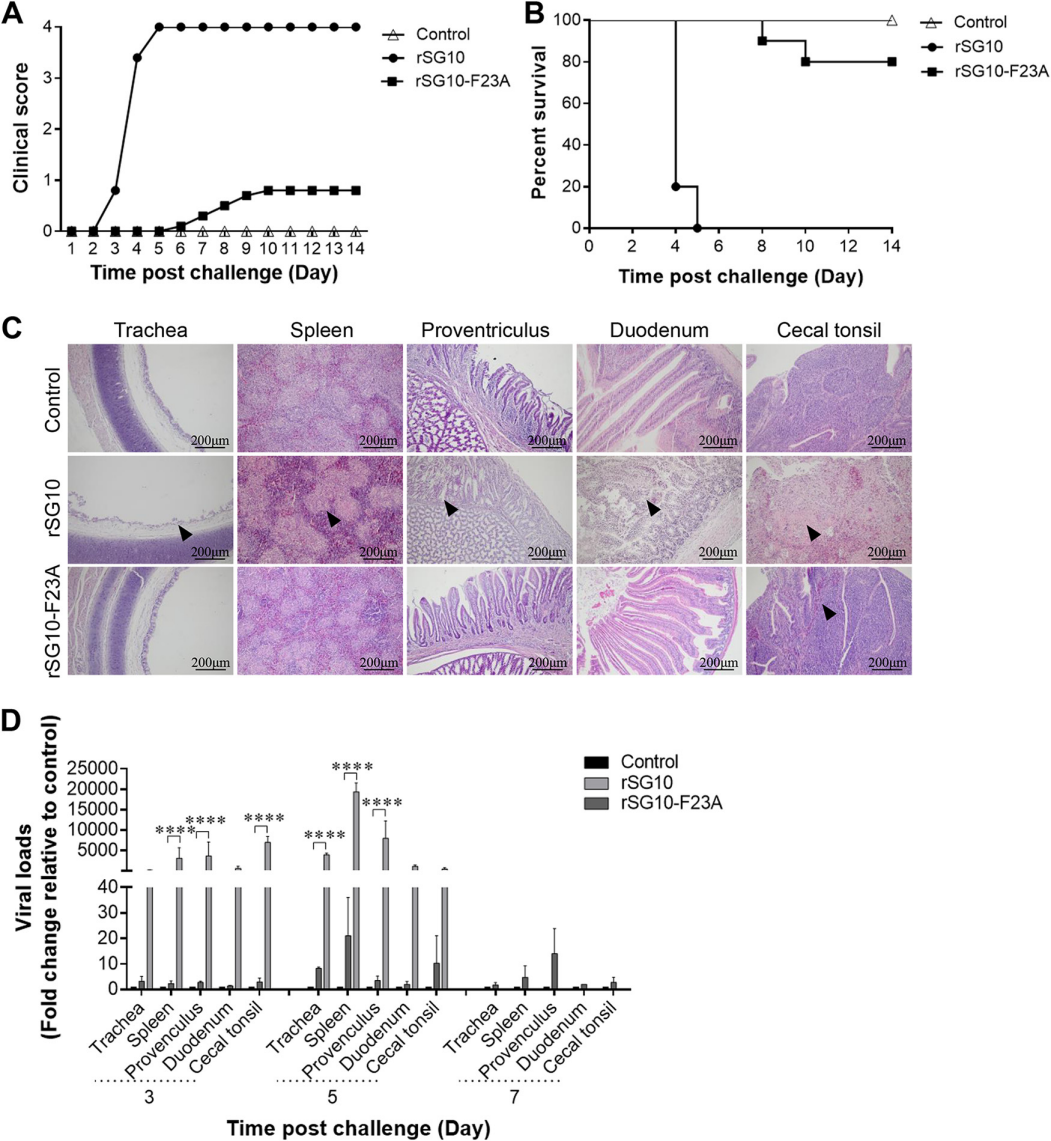
Pathogenicity and replication ability of rNDVs *in vivo*. Three-week-old SPF chickens were inoculated with PBS (uninfected control group) or rNDVs (rSG10 or rSG10-F23A). (A) Clinical symptoms of 10 birds per group were scored daily during the 14-day observation according to the following standard: healthy, 0; sick, 1; wing drop, torticollis, or lack of coordination, 2; prostration, 3; and death, 4. (B) Survival of the control uninfected, rSG10-infected, and rSG10-F23A-infected groups, determined from 10 birds per group. (C) Tissue histopathology analysis at 5 dpc of 3-week-old SPF chickens that had been inoculated with PBS or rNDVs. Tissues were fixed, embedded in paraffin wax, sectioned into slices, and stained in eosin. The lesions of tissues were observed under light microscopy. Black arrowheads indicate the pathological changes observed in tissues. Scale bars: 200 μm. (D) Birds from different groups were euthanized at 3, 5, or 7 dpc, and the viral loads in different tissues were evaluated by RT-qPCR. The comparative threshold cycle (ΔΔ*C_T_*) method was applied, and the 2^−ΔΔ^*^CT^* value of each tissue from the uninfected control group was set to 1. The 2^−ΔΔ^*^CT^* value of each tissue from the rSG10- and rSG10-F23A-infected groups was normalized to the corresponding value of the uninfected control group. The significance of each difference was analyzed by two-way ANOVA.

Next, the pathogenicity and replication ability of rSG10 and rSG10-F23A were evaluated in 3-week-old specific-pathogen-free (SPF) chickens. Chickens in the rSG10 infection group began to show clinical symptoms at 3 days postchallenge (dpc), and all died by 5 dpc, whereas chickens challenged with rSG10-F23A did not show clinical symptoms until 6 dpc, and only 2 of 10 chickens got sick and died during the 14-day observation period. No clinical signs of disease or death were observed in the uninfected control group (Fig. 2A and B). The pathological damage to NDV target organs caused by rSG10-F23A infection was much less severe than that caused by rSG10 infection. Only hemorrhaging in the lamina propria of cecal tonsils was found in the rSG10-F23A infection group, whereas cilium necrosis in the trachea, lymphocyte emptying in the spleen, mucosal epithelial cell shedding in the proventriculus, intestinal villus necrosis in the duodenum, and lymphocyte necrosis in cecal tonsils were all observed in the rSG10 infection group (Fig. 2C).

The viral loads in target tissues were analyzed using reverse transcription-quantitative PCR (RT-qPCR). The results indicate that at 3 dpc and 5 dpc, the average trachea, spleen, proventriculus, duodenum, and cecal tonsil viral loads in rSG10-F23A-infected chickens were all significantly lower than those in rSG10-infected chickens. At 7 dpc, all chickens in the rSG10 group had died, and there was no significant difference in the viral loads between the rSG10-F23A infection group and the uninfected control group (Fig. 2D). Collectively, these results show that an FPIV L-domain mutation can decrease the virulence and pathogenicity of NDV *in vivo*.

### Mutation of the FPIV L-domain blocks NDV virion and VLP release.

**FIG 3:**
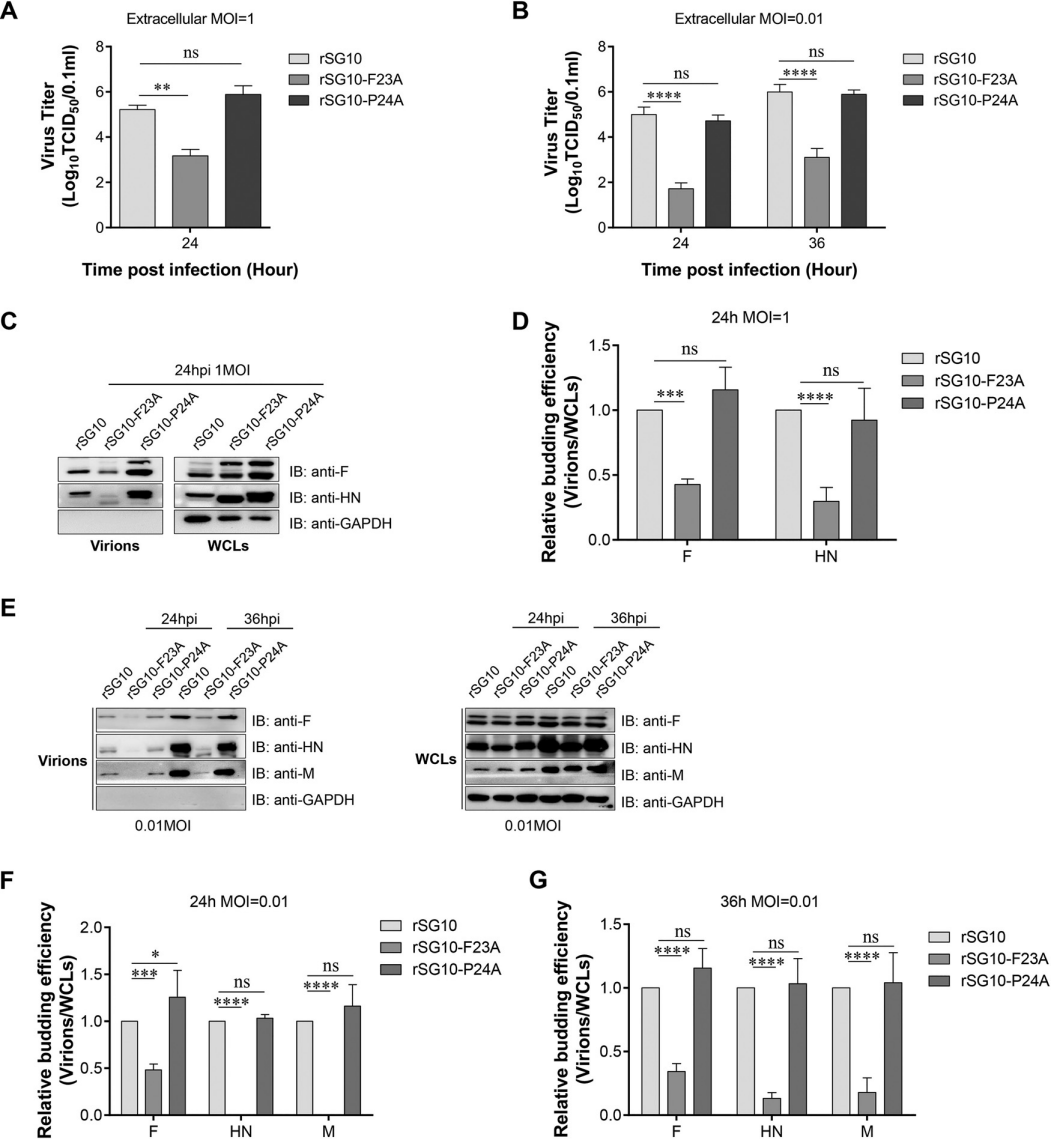
Analysis of the budding efficiency of rNDVs. (A and B) Vero cells were infected with rSG10, rSG10-F23A, or rSG10-P24A at an MOI of 1 (A) or 0.01 (B). At the indicated time points, the extracellular virial titers were determined by titration on the corresponding cells. The significance of each difference was calculated with one-way or two-way ANOVA. (C) Virions were purified via ultracentrifugation. Equal amounts of virions and WCLs were subjected to Western blotting. The HN and M proteins of NDV were detected using specific antibodies. (D) The budding index of rNDVs was defined as the grayscale value of the HN or M protein bands in virions divided by their values in WCLs. The budding efficiency of rSG10 was set to 1, and the budding efficiencies of rSG10-F23A and rSG10-P24A were obtained via comparison with rSG10. The significance of each difference was calculated with two-way ANOVA. The values ± SD from triplicate experiments are shown. (E to G) Virions were purified via ultracentrifugation at the indicated time points. The F, HN, and M proteins of NDV were detected using specific antibodies. The budding efficiency of rNDVs was calculated as described for panel D. The values ± SD from triplicate experiments are shown.

L-domains always play an important role in viral release (26). To study whether the biological characteristic changes of rSG10-F23A were the consequence of budding deficiency caused by the FPIV L-domain mutation, experiments testing the budding efficiency of rNDVs were performed. The budding efficiency of rSG10-P24A was also evaluated. The extracellular viral titers of Vero cells infected with different multiplicities of infection (MOIs) of rNDV were quantified by determining the 50% tissue culture infective dose (TCID_50_). At an MOI of 1, no significant difference was observed between the extracellular viral titers of rSG10-P24A and rSG10, but the extracellular viral titer of rSG10-F23A was significantly lower than that of rSG10 (Fig. 3A). The same experiment was repeated using an MOI of 0.01. The results showed that the extracellular viral titers of rSG10-F23A were lower than those of rSG10 at both 24 h postinfection (hpi) and 36 hpi and that the extracellular viral titers of rSG10-P24A were similar to those of rSG10 (Fig. 3B).

We further evaluated the budding efficiency of rNDVs by using ultracentrifugation and Western blotting as previously described (15). The results reveal that at an MOI of 1, in whole-cell lysates (WCLs) of rSG10-F23A-infected or rSG10-P24A-infected cells, the amounts of NDV fusion (F) protein and hemagglutinin (HA)-neuraminidase (HN) protein were greater than for rSG10-infected cells. However, in the pellets purified from the corresponding cell supernatants, the amounts of the F and HN proteins of rSG10-F23A were significantly lower than those of rSG10, while the amounts of the F and HN proteins of rSG10-P24A were higher than those of rSG10 (Fig. 3C). When the virion-to-WCL ratio of rSG10 was to 100%, the relative budding ratio of rSG10-F23A was 43% when calculated with F protein and 30% when calculated with HN protein. Also, the budding ratio of rSG10-P24A was similar to that of rSG10 (Fig. 3D). The same experiment was repeated using an MOI of 0.01. The results showed that the amounts of the NDV F, HN, and M proteins in WCLs of rNDV-infected cells were similar among the three rNDV strains. But in the pellets, the amounts of the F, HN, and M proteins of rSG10-F23A were significantly lower than for those of rSG10 and rSG10-P24A (Fig. 3E). When the virion-to-WCL ratio of rSG10 was 100%, the relative budding ratio of rSG10-F23A was 34%, 13%, or 18% when calculated with F, HN, or M protein, respectively. And the budding ratio of rSG10-P24A was similar to that of rSG10 (Fig. 3F and G).

**FIG 4:**
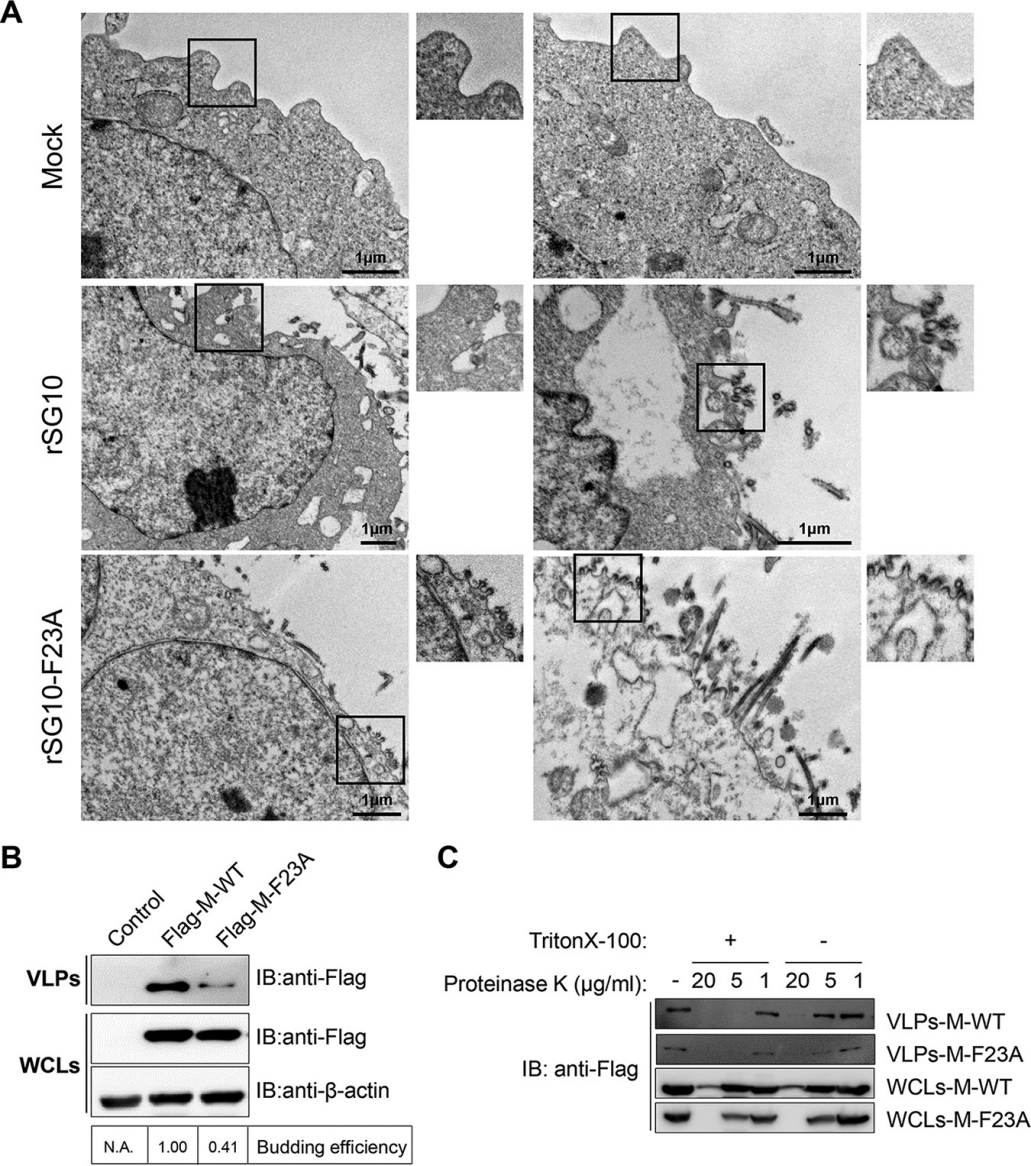
Analysis of the budding efficiency of NDV VLPs. (A) TEM images of thin-sectioned Vero cells that had been infected with an rNDV at an MOI of 0.01. Typical paramyxovirus particles (diameters, 100 to 200 nm) were observed in the plasma membrane. Scale bars = 1 μm. (B) Vero cells were transfected with plasmids expressing Flag-M-WT or Flag-M-F23A. At 36 hpt, the VLPs were purified. Equal amounts of cell lysates and VLPs were subjected to Western blotting (IB). The budding efficiency was calculated as described for Fig. 3D. The assays were performed three times. (C) Collected VLPs and cell lysates were treated with different concentrations (1, 5, or 20 μg/mL) of proteinase K for 30 min in the presence of 1% Triton X-100 or were left untreated. The resulting digestions were subjected to Western blotting.

Finally, to gain more detailed insights into the budding process of rSG10-F23A, the strain that showed significant budding deficiency among experimental strains, we performed a transmission electron microscopy (TEM) analysis of rNDV-infected Vero cells. Overall, areas in which budding virions accumulated were observed more commonly in the cells infected with rSG10-F23A (Fig. 4A). Given the smaller amount of viral particles we reported above in the supernatants of rSG10-F23A-infected cells, we suspect that a great mass of rSG10-F23A progeny virions were arrested at the plasma membrane during budding. Together, these results indicate that in cells infected at either a high or low MOI, rSG10-F23A exhibited a budding deficiency, which was the consequence of progeny virions trapped in the budding zone.

The wild-type M protein of NDV rSG10 (M-WT) and an M-WT with Phe23 replaced by Ala (M-F23A) were appended with an N-terminal Flag tag and transfected into Vero cells to produce VLPs. At 36 h posttransfection (hpt), VLPs were collected from the cell supernatants, pelleted through a sucrose cushion, and analyzed by using a Western blot assay. The results indicate that VLPs were produced more sparsely in the M-F23A-transfected cells than in the M-WT-transfected cells (Fig. 4B), which is consistent with our previous observations in cells infected with these rNDV strains. A protease protection assay was performed to determine if the M protein purified from the supernatants of transfected cells was membrane bound. Collected VLPs and WCLs were incubated with different concentrations of proteinase K, and the resulting digestion products were analyzed via Western blotting. As expected, the M proteins within VLPs were largely protected from protease digestion. In support of this conclusion, disrupting the VLPs with Triton X-100 resulted in the digestion of the M protein inside VLPs (Fig. 4C). Collectively, these findings reveal that the budding deficiency observed in rSG10-F23A infection also occurred in cells transfected with M-F23A.

### Interaction between ESCRT components and NDV M protein.

**FIG 5:**
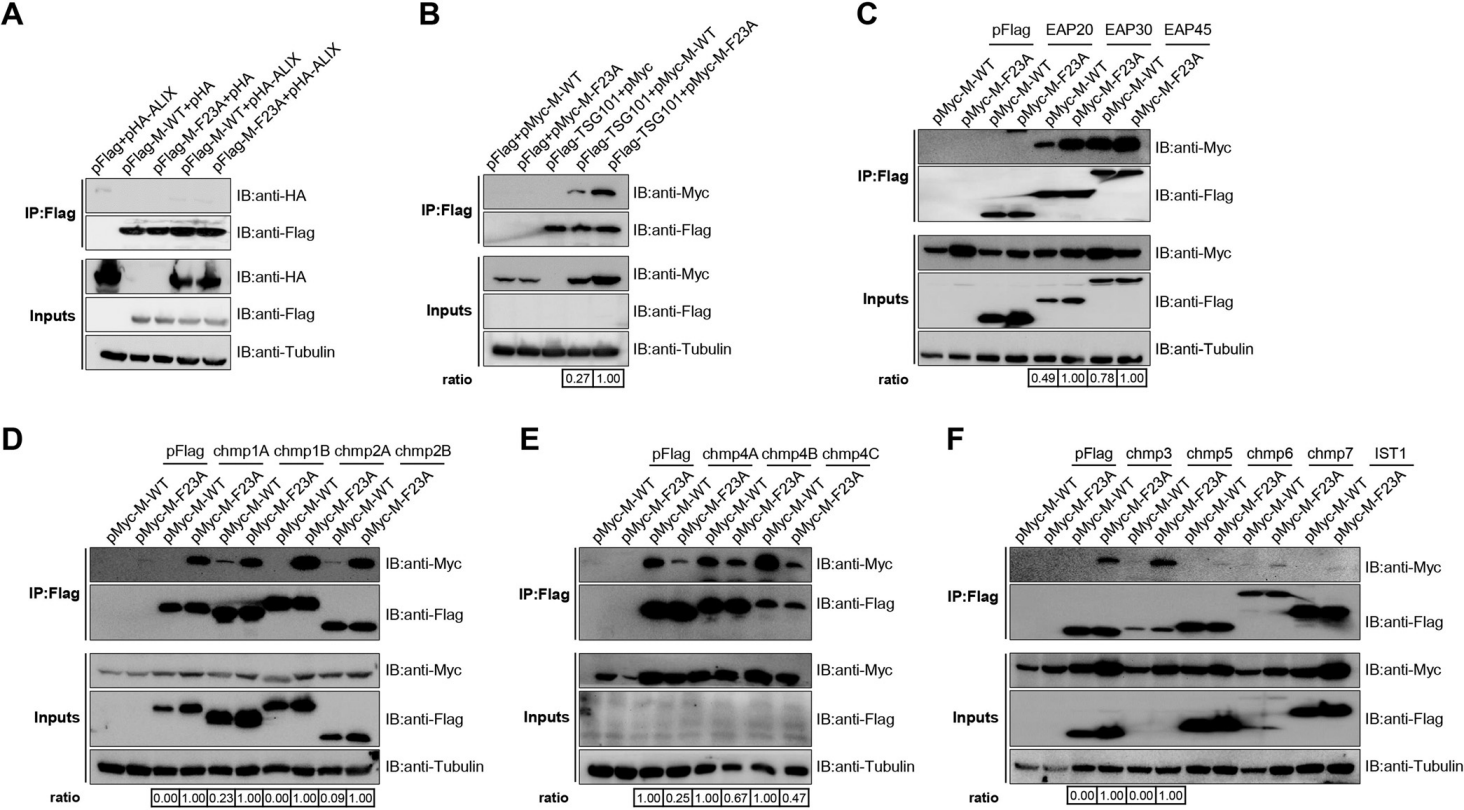
Detection of interaction between ESCRT proteins and the FPIV L-domain. HA-tagged ALIX and Flag-tagged M protein (M-WT or M-F23A) (A) or Myc-tagged M protein (M-WT or M-F23A) and Flag-tagged ESCRT protein (TSG101, EAP20, EAP30, EAP45, CHMP1A, CHMP1B, CHMP2A, CHMP2B, CHMP3, CHMP4A, CHMP4B, CHMP4C, CHMP5, CHMP6, CHMP7, or IST1) (B to F) were cotransfected into HEK293T cells. At 48 hpt, the cells were lysed and immunoprecipitated by using an anti-Flag M2 affinity gel. The assays were performed twice (A and B) or three times (C to F). The resulting immunoprecipitates together with cell lysates were analyzed via Western blotting. The affinity ratio between M protein (M-WT or M-F23A) and each ESCRT component was calculated by dividing the quantity of copurified HA/Myc-tagged protein by the quantity of purified Flag-tagged protein. The affinity ratio of M-WT or M-F23A was set to 1, and the relative affinity ratios of other proteins were obtained by comparison.

A wide range of viruses utilize the L-domain to highjack host ESCRT machinery and facilitate progeny virion release (26). With the hope of uncovering the molecular mechanism behind the budding deficiency of rSG10-F23A, we focused our following studies on exploring the bridging proteins between the FPIV L-domain and ESCRT machinery. The interactions between the NDV M protein (M-WT or M-F23A) and each of 17 pivotal ESCRT machinery proteins were examined. Each ESCRT component was expressed in combination with M-WT or M-F23A in HEK293T cells, and at 48 hpt, the cells were lysed and immunoprecipitated with M2 affinity gels. The results of Western blotting conducted on the immunoprecipitates revealed that among the 17 ESCRT candidates, ALIX, EAP20, CHMP6, CHMP7, and IST1 were unable to be copurified with either M-WT or M-F23A, while CHMP1A, CHMP1B, CHMP3, and CHMP5 could be copurified only with M-F23A. Additionally, TSG101, EAP30, EAP45, CHMP1B, CHMP2B, CHMP4A, CHMP4B, and CHMP4C could be copurified with both M-WT and M-F23A, but only the CHMP4s (CHMP4A, CHMP4B, and CHMP4C) exhibited less interaction with M-F23A than with M-WT (Fig. 5A to F). These results suggest that the FPIV L-domain of M-WT is responsible for efficient recruitment of CHMP4s. The weaker affinity between M-F23A and CHMP4s and the stronger affinity between M-F23A and ESCRT components, including TSG101, EAP30, CHMP1s, CHMP2s, CHMP3, and CHMP5, might work together to influence the phenotype of M-F23A.

### CHMP4s interact with the FPIV L-domain directly.

**FIG 6:**
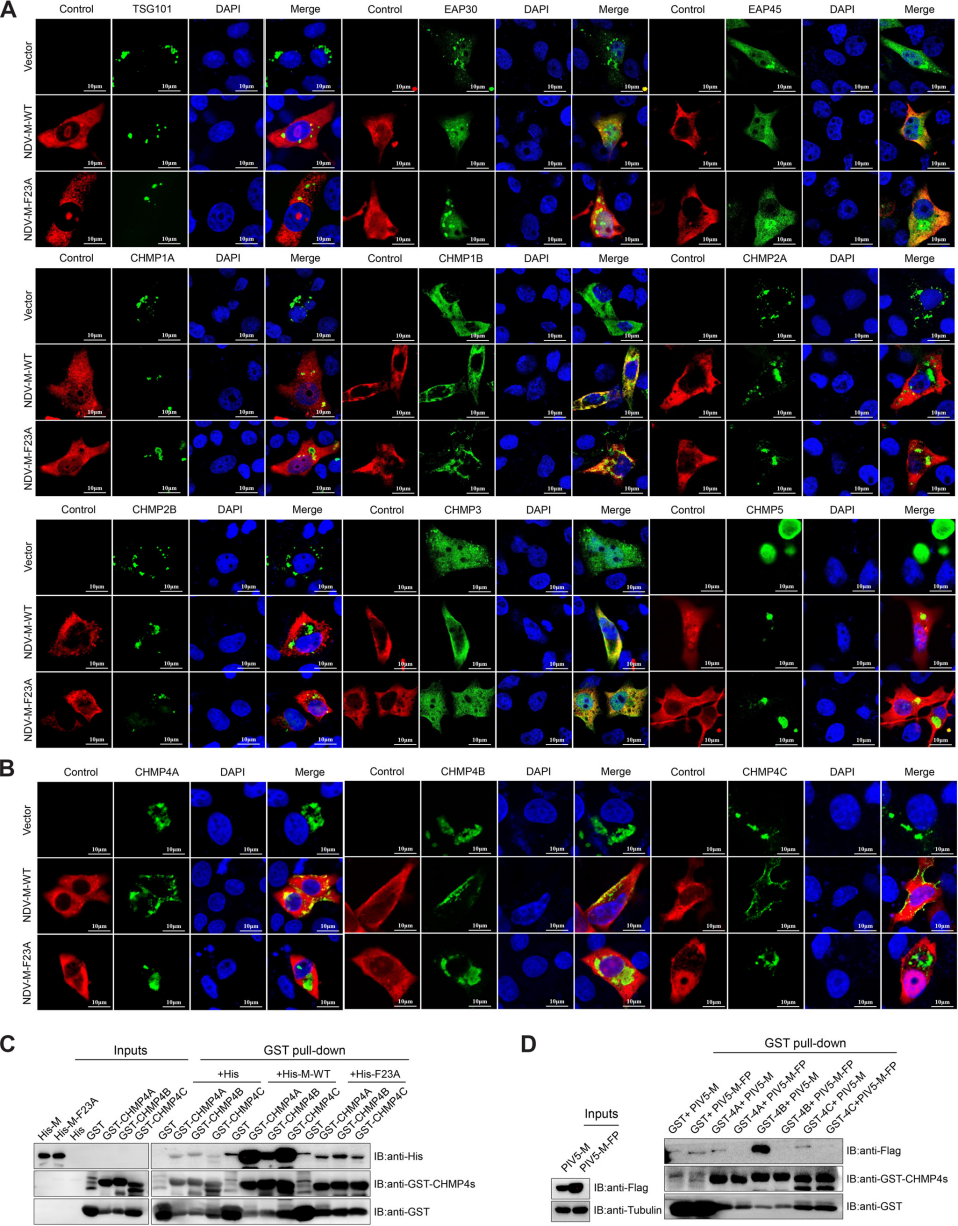
Detection of interaction between the CHMP4s and FPIV L-domain. (A and B) Vero cells were cotransfected with Myc-tagged TSG101, EAP30, EAP45, CHMP1s, CHMP2s, CHMP3, or CHMP5 (A) or CHMP4s (B) and Flag-tagged M protein (M-WT or M-F23A). At 24 hpt, cells were fixed, labeled with antibodies specific for the Myc tag or Flag tag, and incubated with Alexa Fluor 488- and 555-conjugated secondary antibodies. DAPI was used to stain the nuclei. Scale bars = 10 μm. (C and D) GST or GST-tagged CHMP4s were immobilized on glutathione Sepharose 4B and then incubated with lysates of bacteria containing His, His–M-WT, or His–M-F23A (C) or lysates of cells transfected with PIV5-M or PIV5-M-FP (D). The resulting immunoprecipitations were subjected to Western blotting conducted with antibodies specific for the GST, His, and Flag tags. The assays were run in triplicate.

To confirm the coimmunoprecipitation results, M protein (M-WT or M-F23A) and TSG101, EAP30, EAP45, CHMP1s, CHMP2s, CHMP3, CHMP4s, and CHMP5 were cotransfected into Vero cells, and their colocalization was observed by using confocal microscopy. M protein, EAP30, EAP45, CHMP1B, and CHMP3 were observed to be distributed mainly in the cytoplasm, with smaller amounts of them also detected in the nucleoli (Fig. 6A), while TSG101, CHMP1A, CHMP2s, CHMP5, and CHMP4s clustered in clumps in the cytoplasm (Fig. 6A and B). The overexpression of M-WT significantly changed the subcellular location of CHMP4s but not other components of ESCRT machinery, causing them to colocalize with M-WT around the plasma membrane. But the overexpression of M-F23A did not change the subcellular location of CHMP4s, and the CHMP4s protein still aggregated in the cytoplasm as a control (Fig. 6B). Moreover, although some of the interactions between M-WT and CHMP1A, CHMP2A, CHMP3, and CHMP5 were not detected in the coimmunoprecipitation assay, they showed weak colocalization signals in the cytoplasm like M-F23A and CHMP1A, CHMP2A, CHMP3, and CHMP5 (Fig. 6A).

To further clarify if CHMP4s were recruited directly by the FPIV L-domain without the need for accessory proteins, a glutathione *S*-transferase (GST) pulldown assay using GST-tagged CHMP4s or GST and prokaryotically expressed His-tagged M-WT/M-F23A proteins was performed. The results revealed that a large amount of His-tagged M-WT was copurified with GST-tagged CHMP4s (GST-CHMP4s), whereas only a limited amount of His-tagged M-F23A was copurified with GST-CHMP4s (Fig. 6C). Furthermore, a similar experiment using the M protein of PIV5 (PIV5-M) revealed that wild-type PIV5-M could be copurified more efficiently with GST-CHMP4s, especially CHMP4B, than its FPIV L-domain mutant PIV5-M-FP; this finding illustrates that the interaction between the FPIV L-domain and CHMP4s has broad applicability (Fig. 6D). Collectively, these results all confirm that the CHMP4s can interact with NDV M protein directly through the FPIV L-domain.

### Knockdown of CHMP4B inhibits release of NDV particles.

**FIG 7:**
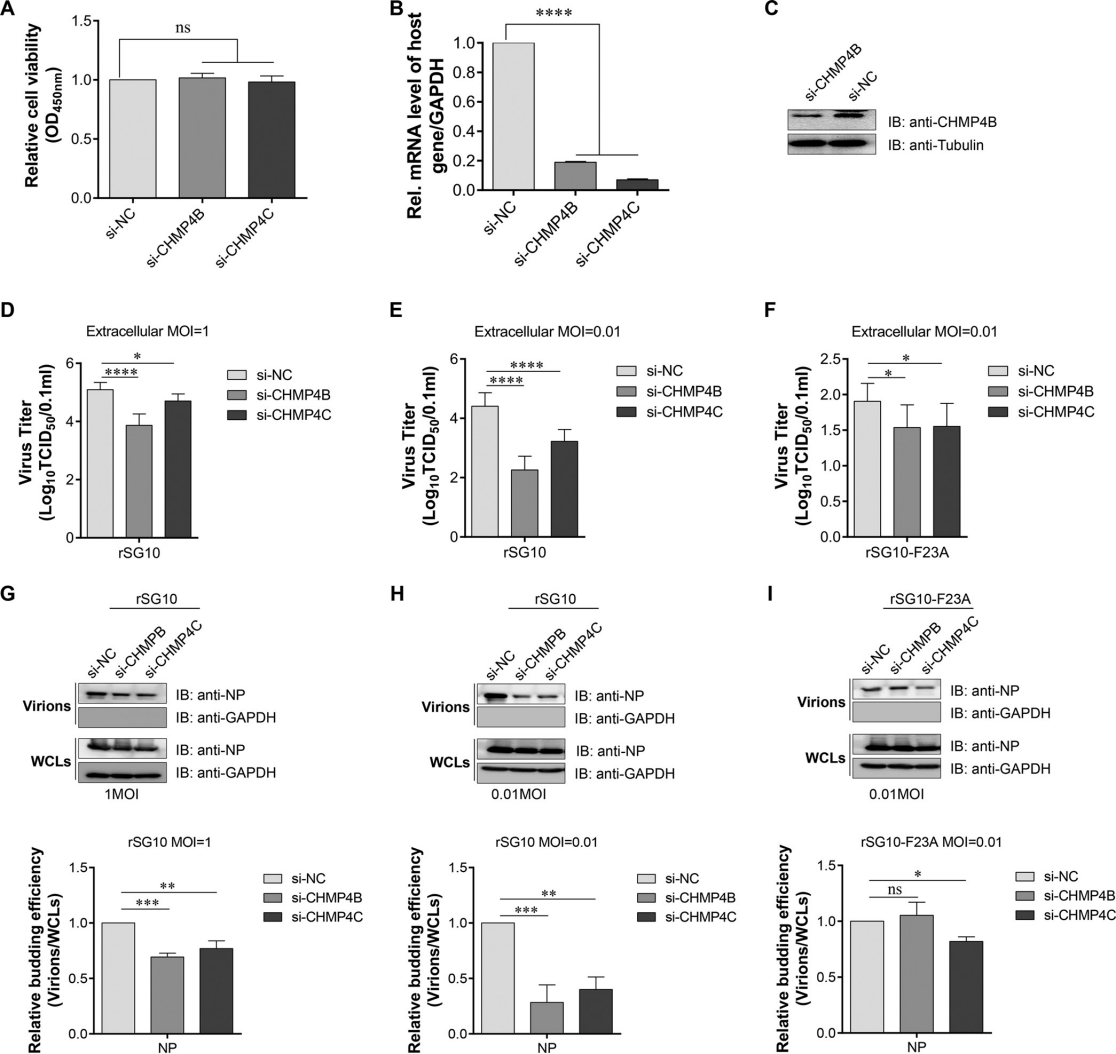
Effects of endogenous CHMP4B knockdown on rSG10 virion release from cells infected at an MOI of 1 or 0.01. (A) Cell viability upon transfection with each indicated siRNA was evaluated via a CCK-8 assay. The value of the si-NC-transfected sample was set to 1. The values of si-CHMP4B- and si-CHMP4C-transfected samples were obtained by comparison with the si-NC-transfected sample. The significance of each difference was calculated by one-way ANOVA. (B) Vero cells were transfected with the indicated siRNA and harvested at 24 hpt for RNA isolation; the resulting RNA was subjected to RT-qPCR to evaluate the percentage of remaining mRNA for CHMP4B or CHMP4C after knockdown. The significance of each difference was calculated with one-way ANOVA. (C) At 48 hpt with the siRNA described above, Vero cells were harvested, and the WCLs were analyzed by Western blotting conducted using specific antibodies against endogenous CHMP4B. (D to F) After transfection with the indicated siRNAs, Vero cells were infected with the indicated rNDV at an MOI of 1 (D) or 0.01 (E and F). At 24 hpi, the extracellular viruses were collected from the infected cells and their titers were determined by titration on corresponding cells. The significance of each difference was calculated with one-way ANOVA. (G to I) The extracellular viruses described for panels D to F were also pelleted through ultracentrifugation. Equal amounts of virions and WCLs were subjected to Western blotting and the budding efficiencies were calculated as described above. The significance of each difference was calculated with one-way ANOVA. The values ± SD from triplicate experiments are shown.

The CHMP4s are the core components of ESCRT-III. CHMP4 depletion significantly affects the release of some viruses (30, 31). To study the involvement of CHMP4s in NDV budding, Vero cells were transfected with small interfering RNA (siRNA) targeting CHMP4B or CHMP4C. No significant drop in cell viability was observed after transfection with any of these siRNAs (Fig. 7A). The knockdown of CHMP4B after siRNA treatment was confirmed at both the mRNA (Fig. 7B) and protein (Fig. 7C) levels, and the knockdown of CHMP4C was confirmed at the mRNA level.

In following experiments, two different infection doses were used to evaluate the budding efficiency of rNDVs after siRNA knockdown. When Vero cells were infected at an MOI of 1, the extracellular viral titer of the CHMP4B or CHMP4C knockdown group was found to be lower than that of the negative-control (NC) group, which illustrates that both CHMP4B and CHMP4C play a role in the release of rSG10 particles (Fig. 7D). To further confirm this finding, an MOI of 0.01 was used and the extracellular viral titers were also quantified; after CHMP4B or CHMP4C knockdown, the extracellular viral titers of rSG10 were about 1 to ~2 logs lower than those of NC-treated cells (Fig. 7E); in contrast, the extracellular viral titers of rSG10-F23A were only half a log lower than those of NC-treated cells (Fig. 7F).

Next, the same volume of the supernatants was collected from corresponding knockdown cells and pelleted through ultracentrifugation. When the cells were infected with rSG10 at an MOI of 1, the amount of nucleoprotein (NP) of the CHMP4B or CHMP4C knockdown group in the pellets was lower than that of the NC group. The budding efficiency of each siRNA knockdown group was calculated as described above, and the results indicated that the budding efficiency of the CHMP4B or CHMP4C knockdown group was 30% or 23% lower than that of the NC group (Fig. 7G). When the cells were infected with rSG10 or rSG10-F23A at an MOI of 0.01, the amount of NP in the pellets collected from rSG10-infected CHMP4B or CHMP4C knockdown cells was lower than that collected from rSG10-infected NC-treated cells. In contrast, the amount of NP in the pellets collected from rSG10-F23A-infected CHMP4B knockdown cells was similar to that collected from rSG10-F23A-infected NC-treated cells. Collectively, our budding efficiency results revealed that a knockdown of CHMP4B caused release defects for the rSG10 strain but not for the rSG10-F23A strain (Fig. 7H and I). These results reveal that CHMP4B plays an important role in the budding of NDV bearing a functional FPIV L-domain.

## DISCUSSION

The FPIV L-domain is position independent, functionally interchangeable with other L-domains, and sensitive to the ectopic expression of dominant negative AAA-ATPase VPS4A (32), so host ESCRT machinery was presumed to participate in the release of FPIV L-domain-harboring viruses. Previous studies revealed that the FPIV L-domain could not interact with ALIX (15), an important accessory protein of the ESCRT machinery that links the ESCRT-III and ESCRT-I complexes. Because the interaction partners of the FPIV L-domain were still unknown, we conducted the present study using NDV to obtain a detailed description of the FPIV L-domain and identify the main factor of ESCRT components that are recruited by this L-domain.

Two rNDVs with different single amino acid mutations on the ^23^FPIV^26^ L-domain were rescued, but growth characteristic changes were observed only in the virus with an alteration of amino acid residue F23 (i.e., no such changes were observed in the virus with an alteration of amino acid residue P24). Previous work revealed that a recombinant PIV5 strain containing a single amino acid mutation at P21 (^20^FPIV^23^ L-domain) was barely viable (15), which suggests that the key amino acid position of the FPIV L-domain might be virus specific. Few studies have explored the effects of the L-domain on virus replication *in vivo*. In this study, a pathogenicity assay was conducted with 3-week-old SPF chickens. rSG10-F23A was found to have attenuated pathogenicity and a lower replication ability compared with those of rSG10, which is consistent with the corresponding *in vitro* results.

L-domains always play a role in virus release. L-domain mutations in human immunodeficiency virus (HIV) Gag protein resulted in delayed virion release and VLP accumulation on the plasma membrane (33). L-domain mutations in Ebola virus VP40 (eVP40) protein, which disrupt the eVP40-ITCH interaction, also prevented the budding of eVP40 VLPs (34). Mutations of the PPPY L-domain on the vesicular stomatitis virus (VSV) M protein blocked virus budding in the late stage and also reduced the progeny virion yield (35). Therefore, we focused our study on the budding stage of NDV as a potential explanation for the growth defects exhibited by rSG10-F23A. rSG10-P24A was used as an internal control. We observed that the extracellular viral titers and virion amounts of rSG10-F23A were significantly lower than those of rSG10. Further study using electron microscopy revealed that cell-associated virions appeared more frequently in rSG10-F23A-infected cells than in rSG10-infected cells. Similar phenomena were also observed in 293T cells that were cotransfected with HIV-1 DNA and dominant negative DsRed-CHMP4B, suggesting that these viruses were arrested at the late stage of viral assembly (36). Together, these results demonstrate that the FPIV L-domain contributes to the release of NDV particles, which affects the growth of NDV.

These findings left the question of why a mutation of the FPIV L-domain affects NDV budding. Previously, host ESCRT machinery was proven to participate in the release of various viruses through its direct interaction with viral L-domains (17). Thus, we hypothesized that the FPIV L-domain also hijacks the host ESCRT machinery by interacting with its components. NDV M proteins are sufficient to form and release VLPs from transfected cells in the absence of other viral proteins, demonstrating that NDV M proteins contain adequate information to mediate membrane fission (32). Because we found that a single amino acid mutation of the FPIV L-domain could prevent the budding of NDV VLPs formed by M protein alone, we used exogenously expressed M-WT and its FPIV L-domain mutant M-F23A as bait to identify potential interactions between the FPIV L-domain and mammalian ESCRT proteins. We first tested ALIX (37) and TSG101 (38), the binding partners of the YPx_(1, 3)_L L-domain and P(T/S)AP L-domain, respectively, to establish this approach. Surprisingly, the results indicated that TSG101 was capable of interacting with both M-WT and M-F23A. TSG101 is the core component of the ESCRT-I complex; it not only is required for the budding of P(T/S)AP L-domain-containing virus but also is known to participate in the entry and replication of viruses such as classical swine fever virus (CSFV) and Kaposi’s sarcoma-associated herpesvirus (KSHV) (39, 40). In this study, TSG101 was identified as a binding partner of NDV M protein; its function in NDV’s life cycle was worthy of further study. In addition to TSG101, the ESCRT-II components EAP30 and EAP45 and the ESCRT-III components CHMP1A, CHMP1B, CHMP2A, CHMP2B, CHMP3, and CHMP5 also had the ability to interact with the NDV M protein, but, like for TSG101, the intensity of their interaction with M-F23A was much stronger than with M-WT. One possible reason for this phenomenon is that mutation of the FPIV L-domain weakened the existing ways by which the NDV M protein recruits the ESCRT machinery but enhanced the alternative ones. Alternatively, mutation of the FPIV L-domain may have promoted the structural affinity of the NDV M protein for those ESCRT proteins. Additional research is still needed to explain this observation.

In contrast, the CHMP4s (CHMP4A, CHMP4B, and CHMP4C) were found to exhibit the opposite interaction intensity from the other tested ESCRT components; i.e., the amount of M-WT that was copurified with the CHMP4s was larger than the amount of M-F23A copurified with them. Further experiments confirmed this finding and proved that the CHMP4s could directly bind to the NDV M protein through the FPIV L-domain without the involvement of intermediate proteins. Furthermore, the CHMP4 subcellular locations were changed to the plasma membrane after the overexpression of M-WT but not of M-F23A. To some extent, this result suggests that the CHMP4s could be recruited by the NDV M protein through the FPIV L-domain to the budding site of NDV.

ESCRT-III is in charge of multiple membrane fission processes (21). In most cases, bridging factors are needed to a build physical connection between ESCRT-III and the targeting factors. In mammalian cells, these bridging factors include ESCRT-I, ESCRT-II, and Bro1 domain-harboring proteins like ALIX (41). In this study, the ESCRT-III complex CHMP4s were confirmed to directly bind to the NDV M protein via the FPIV L-domain, and this interaction did not rely on TSG101, ESCRT-II, or ALIX acting as an assembly factor. However, because the collaboration between host proteins is complicated, some unidentified assembly factors of CHMP4s not analyzed in this research, which act to assist the FPIV L-domain with the recruitment of CHMP4s, could exist. More studies are still needed.

The CHMP4s contribute to the budding of various viruses. For example, herpes simplex virus 1 (HSV-1) expropriates the CHMP4s in infected cells for scission of the inner nuclear membrane to export progeny virions (21). Additionally, late in the HIV-1 assembly stage, the CHMP4s are recruited to form transient membrane-associated lattices to facilitate host-virus membrane fission (42, 43). To determine whether the CHMP4s play a key role in the budding stage of the NDV life cycle, we performed siRNA-mediated knockdowns of CHMP4B and CHMP4C. CHMP4A was not analyzed in this experiment because its mRNA level in Vero cells was too low to be detected. The knockdown assay results indicated that among the tested candidates, both CHMP4B and CHMP4C contributed to the release of rSG10, which was similar to the budding of HIV-1 (38). Previous research conducted using the NDV F48E9 strain illustrated that knockdown of CHMP4B protein or overexpression of dominant negative CHMP4B in DF-1 cells significantly restricted growth of the F48E9 strain and decreased the extracellular viral titers of F48E9-infected cells, which suggest that CHMP4B is involved in the growth of NDV, especially in the late stage (44). These findings prompt us to consider that the failure of rSG10-F23A to bind CHMP4s, especially CHMP4B, contributes to the budding deficiency exhibited by rSG10-F23A. To further confirm the effect of CHMP4B during NDV budding, establishment of a CHMP4B knockout cell line or codepletion of CHMP4A/B/C will be necessary in a future study.

The interaction between the CHMP4s and NDV M protein did not completely disappear after the introduction of a single amino acid mutation at the F23 site of the ^23^FPIV^26^ L-domain, and the budding process of NDV was not completely restricted by this mutation either. The most likely explanation for these results is that (i) the single amino acid mutation was unable to completely block the FPIV-CHMP4 interaction, (ii) dimers of the NDV M protein on the inner surface of the plasma membrane can drive the release of some particles, or (iii) other budding mechanisms in addition to the FPIV-CHMP4 interaction might be used by NDV. Previous studies showed that respiratory syncytial virus uses a VPS4-independent budding mechanism controlled by Rab11-FIP2 (45). Additionally, AMOTL1 linked the PIV5 M protein to the Nedd4 family ubiquitin ligases to facilitate viral budding (46). In the present study, TSG101, EAP30, EAP45, and many other components of the ESCRT machinery were found to act as binding partners of the NDV M protein. The effects of these interactions on the NDV life cycle are worthy of further study.

In summary, the results of this work suggest that after a key amino acid mutation of the FPIV L-domain, the NDV M protein could not interact efficiently with CHMP4B, thereby causing progeny virions to arrest during budding at the plasma membrane, consequently leading to release and growth defects of the NDV strain. This research not only enriches our current understanding of the FPIV L-domain function, providing a new target for the attenuation of NDV and other paramyxoviruses, but also reveals a main factor of host ESCRT machinery which is recruited by FPIV L-domain, thus laying the foundation for subsequent studies on the FPIV L-domain. Further research is still needed to reveal how the interaction between the FPIV L-domain and CHMP4B facilitates the budding of NDV.

## MATERIALS AND METHODS

### Cells and viruses.

Vero cells (an African green monkey kidney cell line), HEK293T cells (a human embryonic kidney cell line), and BSR T7/5 cells (a baby hamster kidney cell line stably expressing T7 RNA polymerase) were cultured in Dulbecco’s modified Eagle’s medium (DMEM; Gibco, Grand Island, NY, USA) with 10% fetal bovine serum (FBS; Gibco) and maintained in DMEM with 2% FBS at 37°C in a 5% CO_2_ incubator (Thermo Forma, Marietta, OH, USA). The recombinant NDV strain rSG10 was preserved in our lab, and rSG10-F23A and rSG10-P24A were recovered from BSR T7/5 cells using the NDV rSG10 reverse genetics system.

### Plasmids.

The plasmids pOK-rSG10, pCI-NP, pCI-P, and pCI-L have been described previously (47). The full-length plasmids pOK-rSG10-F23A and pOK-rSG10-P24A were generated via single-site mutation on plasmid pOK-rSG10. cDNA encoding the rSG10 M protein or an M protein with a single amino acid mutation on the F23 site was generated by PCR using cDNA from pOK-rSG10 or pOK-rSG10-F23A as a template and subcloned into the eukaryotic expression vector pRK5-Flag or pCMV-Myc to form pFlag-M-WT and pFlag-M-F23A or pMyc-M-WT and pMyc-M-F23A, respectively. The plasmids pRK5-Flag-TSG101, pRK5-Flag-EAP20, pRK5-Flag-EAP30, pRK5-Flag-EAP45, pRK5-Flag-CHMP1A, pRK5-Flag-CHMP1B, pRK5-Flag-CHMP2A, pRK5-Flag-CHMP2B, pRK5-Flag-CHMP3, pRK5-Flag-CHMP4A, pRK5-Flag-CHMP4B, pRK5-Flag-CHMP4C, pRK5-Flag-CHMP5, pRK5-Flag-CHMP6, pRK5-Flag-CHMP7, pRK5-Flag-IST1, and pRK5-Flag-PIV5-M were generated by cloning the open reading frame (ORF) of each gene into the eukaryotic expression vector pRK5-Flag. pRK5-Flag-PIV5-M-FP was generated via multiple-site mutation on plasmid pRK5-Flag-PIV5-M. The plasmid pRK5-HA-ALIX was generated by cloning the ORF of ALIX with an HA tag at its N terminus into the vector pRK5. The plasmids pCMV-Myc-TSG101, pCMV-Myc-CHMP4A, pCMV-Myc-CHMP4B, and pCMV-Myc-CHMP4C were generated by cloning the ORF of each gene into the vector pCMV-Myc. The plasmids pGEX-GST-CHMP4A, pGEX-GST-CHMP4B, and pGEX-GST-CHMP4C were generated by cloning the ORF of each gene into the prokaryotic expression vector pGEX-6p-1. The plasmids pET32A-His-M-WT and pET32A-His-M-F23A were generated by cloning the ORF of each gene into the prokaryotic expression vector pET32a.

### Antibodies.

Mouse monoclonal antibody against M protein, mouse polyclonal antibody against nucleoprotein (NP), and chicken polyclonal antibody against HN protein were prepared in our laboratory. Monoclonal antibodies specific to the Myc, Flag, HA, and His tags, β-actin, and tubulin used in Western blotting assays and the Alexa Fluor 488- and 555-conjugated secondary antibodies against mouse and rabbit used for indirect immunofluorescence assays (IFAs) were all purchased from Cell Signaling Technology (Beverly, MA, USA). Monoclonal antibody specific to the GST tag was purchased from Solarbio (Beijing, China). The secondary anti-mouse, anti-rabbit, or anti-chicken antibodies used for Western blotting assays were purchased from Bioss Biotechnology (Beijing, China). Anti-Flag M2 magnetic beads were purchased from Sigma-Aldrich (St. Louis, MO, USA). Rabbit polyclonal antibody against CHMP4B (A7402) was purchased from Abclonal (Wuhan, China). Rabbit polyclonal antibody against TSG101 (ab125011) was purchased from Abcam (Cambridge, UK).

### Animals and ethics statement.

SPF embryonated chicken eggs and SPF chickens were purchased from Beijing Boehringer Ingelheim Vital Biotechnology Co., Ltd. (Beijing, China). The experimental protocols used in this study were approved by the Beijing Administration Committee of Laboratory Animals under the leadership of the Beijing Association for Science and Technology (approval no. SYXK [Jing] 2018-0038) and the Ethical Censor Committee at China Agricultural University (CAU; approval no. 2021066).

### Cell infection.

Vero cells were infected with NDV at an MOI of 1 or 0.01. After absorption for 1 h at 37°C, unattached viruses were removed via three washes with phosphate-buffered saline (PBS). Maintenance medium was then added to the wells, and the cells were continuously cultured in the 5% CO_2_ incubator. Samples were harvested at specific postinfection time points. Viral titers on corresponding cells were determined by the TCID_50_ method as described previously (47). Two independent experiments were performed in triplicate.

### Plasmid and siRNA transfection.

HEK293T or Vero cells were grown on plates to 70% to 80% confluence and then transfected with the indicated plasmids using StarFect high-efficiency transfection reagent (GeneStar, Beijing, China) in accordance with the manufacturer’s instructions. At the indicated posttransfection time points, cells were collected for use in further studies.

For siRNA knockdown, Vero cells were transfected with the indicated siRNA using RNAi-Mate (GenePharma, Shanghai, China) in accordance with the manufacturer’s instructions. All siRNA duplexes used in this study were designed and synthesized by GenePharma (Shanghai, Beijing). The sequences of the siRNAs used in this research are listed in Table 1. At 24 hpt, cells were retransfected with the corresponding siRNA, and at 48 hpt, cells were infected with rSG10 or rSG10-F23A at different MOIs.

**TABLE 1:** siRNA duplexes used in this study

siRNA name	Sequence	Use
si-NC-antisense	5′-ACGUGACACGUUCGGAGAATT-3′	CHMP4B knockdown
si-CHMP4B-sense	5′-GGAGGAGCUAGACAAGAAUTT-3′	
si-CHMP4B-antisense	5′-AUUCUUGUCUAGCUCCUCCTT-3′	CHMP4C knockdown
si-CHMP4C-sense	5′-GGCAAUGAAAGCAGUUCAUTT-3′	
si-CHMP4C-antisense	5′-AUGAACUGCUUUCAUUGCCTT-3′	Negative control
si-NC-sense	5′-UUCUCCGAACGUGUCACGUTT-3′	

### Virion/VLP purification and Western blotting.

Virions/VLPs were collected at specific postinfection/posttransfection time points and purified as described previously (48). The resulting pellets were collected from the bottom and dissolved in 100 μL of Tris-EDTA buffer solution. Corresponding cell lysates were prepared by using a ProteinExt mammalian total protein extraction kit (TransGen, Beijing, China). The virions/VLPs together with cell lysates were then analyzed via Western blotting as described previously (49).

### Protease protection assay.

VLPs purified from cell supernatants were separated into two groups. Each group of VLPs and its corresponding WCLs were treated as described previously (50). The samples were further analyzed via Western blotting.

### TEM.

Vero cells were infected with rNDV at an MOI of 0.01, and at 32 hpi, the cells were fixed, dehydrated, embedded in epoxy resin, and cut into 70-nm sections as described previously (51). These sections were examined on a JEM-1230 TEM (JEOL, Tokyo, Japan). Virions were identified on the basis of shape and size.

### Coimmunoprecipitation.

Transfected HEK293T cells were harvested at 48 hpt and then lysed in Pierce immunoprecipitation (IP) lysis buffer (Thermo) to obtain WCLs. After centrifugation at 14,000 × *g* for 10 min, the resulting supernatants were cleared with mouse IgG agarose (Beyotime Biotechnology, Shanghai, China) for 2 h and then incubated with anti-Flag M2 affinity gel (Sigma-Aldrich) overnight at 4°C. The resulting immunoprecipitates and cell lysates were further analyzed via Western blotting.

### IFA.

Vero cells were seeded on cell slides in 24-well plates and transfected with the indicated plasmids; cell samples were then harvested at 24 hpt. These samples were fixed in immunol staining fix solution, permeabilized in immunostaining permeabilization buffer, and blocked with blocking buffer (Beyotime Biotechnology) at room temperature. The cells were subsequently incubated with primary antibodies overnight at 4°C. After being washed three times with PBS, the cells were incubated with secondary antibody at room temperature for 1 h. The nuclei were stained with 4′,6-diamidino-2-phenylindole (DAPI; Sigma-Aldrich) at room temperature for 15 min. Fluorescent images were obtained with a Nikon A1 confocal microscope (Nikon, Tokyo, Japan). The resulting images were analyzed by ImageJ.

### GST pulldown assay.

Escherichia coli BL21-DE3 strains (TransGen) containing pGEX-6p-1, pGEX-GST-CHMP4A, pGEX-GST-CHMP4B, or pGEX-GST-CHMP4C were grown in lysogeny broth medium at 37°C for 4 to 6 h. Isopropyl-β-d-thiogalactopyranoside (IPTG) was then added to the cell culture at a final concentration of 1 mM, and cells were further nourished at 16°C for 14 to 16 h. Finally, the cells were pelleted and lysed on ice with an ultrasonic cell disrupter system. The resulting lysates were clarified by centrifugation and incubated with glutathione Sepharose beads (GE Healthcare, Uppsala, Sweden) at room temperature for 2 h. After three washes with PBS, the cell lysates from rSG10- or rSG10-F23A-infected cells, bacterial lysates from pET32A-, pET32A-His-M-WT-, or pET32A-His-M-F23A-containing E. coli BL21-DE3 cells, or cell lysates from PIV5-M- or PIV5-M-FP-transfected cells were added to the beads and incubated at 4°C overnight. After three washes with PBS, the immunoprecipitates were eluted with Tris-Cl (50 mM; pH 8.0) containing 20 mM reduced glutathione. The bound protein was then subjected to Western blotting.

### Cell viability assay.

Vero cells were seeded in a 96-well plate to 70% to 80% confluence and transfected with siRNA in accordance with the manufacturer’s instructions. The cytotoxicity of each siRNA on Vero cells was analyzed by using a Cell Counting Kit-8 (CCK-8) (Beyotime Biotechnology). Briefly, at 48 hpt, CCK-8 solution was added to each sample, and the cells were incubated at 37°C for another 3 h. The sample absorbance was measured by a microplate reader at 450 nm. Three independent experiments were performed in triplicate.

### RT-qPCR.

Total RNA was extracted using a total RNA isolation kit (FORE GENE, Chengdu, China) and reverse transcribed into cDNA. RT-qPCR was performed on this cDNA by using M5 HiPer real-time PCR supermix (Mei5bio, Beijing, China). Three independent experiments were performed in duplicate. The mRNA levels of the target genes of each sample were detected and normalized to the mRNA level of the β-actin/glyceraldehyde-3-phosphate dehydrogenase (GAPDH) gene of the sample, and the value of the control group was set to 1.

### MDT and ICPI.

The MDTs and ICPIs of NDV strains were evaluated as described before (47). The virulence of each NDV strain was ranked in accordance with the following standard: virulent strains, MDT of <60 h and ICPI of 1.50 to 2.00; moderately virulent strains, MDT of 60 to 90 h and ICPI of 0.70 to 1.50; and avirulent strains, MDT of >90 h and ICPI of 0.00 to 0.70.

### Pathogenicity in 3-week-old chickens.

The experiment performed to determine the pathogenicity of rSG10 and rSG10-F23A was conducted in 3-week-old chickens. SPF chickens were separated randomly into three groups and inoculated through the oculo-nasal route with 100 μL of PBS or a dose of 10^5^ 50% egg lethal doses (ELD_50_) of virus per bird. During the 14-day observation period, clinical symptoms and mortality were recorded, and each chicken was scored using the following scale: healthy, 0; sick, 1; wing drop, paralysis, torticollis, or lack of coordination, 2; prostration, 3; and death, 4. At 3, 5, and 7 days postinfection (dpi), two birds from each group were euthanized, and trachea, spleen, proventriculus, duodenum, and cecum tonsil samples were collected for the detection of viral loads by RT-qPCR. Tissues from chickens euthanized at 5 dpi were fixed and used for histopathology analysis.

### Data analysis.

All data were analyzed with GraphPad Prism software version 5.0 (GraphPad Software Inc., San Diego, CA, USA). To evaluate the significance of differences, one-way and two-way analyses of variance (ANOVA) were used. Statistical significance is indicated in the figures as follows: ns, not significant; *, *P < *0.05; **, *P < *0.01; ***, *P < *0.001; and ****, *P < *0.0001.
